# Purification and Functional Characterisation of Rhinocerase, a Novel Serine Protease from the Venom of *Bitis gabonica rhinoceros*

**DOI:** 10.1371/journal.pone.0009687

**Published:** 2010-03-12

**Authors:** Sakthivel Vaiyapuri, Robert A. Harrison, Andrew B. Bicknell, Jonathan M. Gibbins, Gail Hutchinson

**Affiliations:** 1 School of Biological Sciences, University of Reading, Reading, United Kingdom; 2 The Alistair Reid Venom Research Unit, Liverpool School of Tropical Medicine, Liverpool, United Kingdom; 3 Blood Transfusion Research Group, King Saud University, Riyadh, Saudi Arabia; Griffith University, Australia

## Abstract

**Background:**

Serine proteases are a major component of viper venoms and are thought to disrupt several distinct elements of the blood coagulation system of envenomed victims. A detailed understanding of the functions of these enzymes is important both for acquiring a fuller understanding of the pathology of envenoming and because these venom proteins have shown potential in treating blood coagulation disorders.

**Methodology/Principal Findings:**

In this study a novel, highly abundant serine protease, which we have named rhinocerase, has been isolated and characterised from the venom of *Bitis gabonica rhinoceros* using liquid phase isoelectric focusing and gel filtration. Like many viper venom serine proteases, this enzyme is glycosylated; the estimated molecular mass of the native enzyme is approximately 36kDa, which reduces to 31kDa after deglycosylation. The partial amino acid sequence shows similarity to other viper venom serine proteases, but is clearly distinct from the sequence of the only other sequenced serine protease from *Bitis gabonica*. Other viper venom serine proteases have been shown to exert distinct biological effects, and our preliminary functional characterization of rhinocerase suggest it to be multifunctional. It is capable of degrading α and β chains of fibrinogen, dissolving plasma clots and of hydrolysing a kallikrein substrate.

**Conclusions/Significance:**

A novel multifunctional viper venom serine protease has been isolated and characterised. The activities of the enzyme are consistent with the known *in vivo* effects of *Bitis gabonica* envenoming, including bleeding disorders, clotting disorders and hypotension. This study will form the basis for future research to understand the mechanisms of serine protease action, and examine the potential for rhinocerase to be used clinically to reduce the risk of human haemostatic disorders such as heart attacks and strokes.

## Introduction

Snake venom serine proteases exert their effects through the ability to disrupt the blood coagulation system of envenomed prey and victims [1]. They are found mainly in the venoms of snakes belonging to the viperidae family with a few occurring in members of the elapidae, colubridae and hydrophidae families [2]. Viper venom serine proteases (VVSPs) affect various stages of the blood coagulation system. These include pro-coagulant enzymes such as thrombin-like enzymes which clot fibrinogen (fibrinogenolytic), factor V activators, kininogenases and platelet aggregators, and anti-coagulant enzymes such as fibrinolytic enzymes, plasminogen activators and protein C activators [3]. Characterised VVSPs belong to the S1 family within the SA clan of serine proteases; they contain 230–236 amino acids with molecular masses between 28kDa and 38kDa due to their varying glycosylation contents and have a chymotrypsin-like structural fold [2]. VVSPs typically exhibit 60–90% sequence identity with each other and 30–35% identity with mammalian trypsin and thrombin [4], [5]. Despite their high sequence similarity, there is considerable variation in the functions of the VVSPs. This functional diversity is conferred therefore by less than 40% of the total VVSP sequence.

Understanding the relationships between the sequences, structures and functions of VVSPs is vital for understanding the specificity of these enzymes and their effects on prey and victims. The sequences of over 100 VVSPs are known, but only 4 crystal structures have been determined [6]–[8]. Detailed functional information is also limited; our preliminary analysis showed that no functional information is available for the majority (80%) of VVSP sequences. From the venom of West African gaboon viper *Bitis gabonica rhinoceros* only one serine protease had been sequenced and no functional analysis was carried out on this protein, although intriguingly the sequence lacks the classical serine protease catalytic triad [9]. The existence of other serine proteases [10], [11] in the venom of *B. g. rhinoceros,* including gabonase, a clotting serine protease [12] has been mentioned in the literature. To further understanding of this family of venom enzymes there is a need for detailed functional characterisation of specific VVSPs. As a step towards this goal here we report the purification, partial amino acid sequencing and preliminary functional characterisation of a novel serine protease from the venom of *B. g. rhinoceros*.

## Materials and Methods

*Materials used*- Lyophilized venom of *B. g. rhinoceros* was obtained from a pool of four specimens maintained in the Liverpool School of Tropical Medicine, Liverpool, UK. Protein molecular weight markers, polyvinylidene fluoride (PVDF) membranes and aliphatic ampholytes were from Bio-Rad (Hemel Hempstead, UK). The low molecular weight column calibration kit, enhanced chemiluminescence (ECL) reagents and ECL glycoprotein detection module were from GE Healthcare (Amersham, UK). N Glycosidase F enzyme was from Roche Diagnostics Limited (West Sussex, UK) and trypsin and thrombin were from Promega (Southampton, UK). Fluorescent substrates, PROTIA- Proteoprep Immunoaffinity albumin and IgG depletion kit and all other chemicals used in this research were analytical grade from Sigma Aldrich (Poole, UK).

*SDS-PAGE and Immuno blotting*- Reducing SDS-PAGE, staining and immuno blotting onto polyvinylidine difluoride (PVDF) membrane were all performed using standard techniques [13].

*Serine protease assay*- Serine protease activity of venom and purified proteins was measured using a fluorescent substrate, Nα-benzoyl-L-arginine 7-amido-4-methylcoumarin.HCl (Arg-AMC) (B7260, Sigma Aldrich) as previously described [14]. Venom or purified proteins in 0.1M Tris-HCl pH7.4 were mixed with various concentrations of Arg-AMC along with trypsin and thrombin as positive controls and incubated at 37°C. The amount of 7-amido-4-methylcoumarin (AMC) released was measured at different time points by spectrofluorimeter (FLUOstar OPTIMA, Offenburg, Germany) at an excitation wavelength of 366 nm and an emission wavelength of 460 nm. The kinetic parameters were calculated from Lineweaver-Burk plots. The results are represented by *Km, Vmax, Kcat* and *Kcat/Km* values. All measurements were performed in triplicate.

*Protein purification*- A serine protease present in the venom was purified by liquid phase isoelectric focussing using a micro rotofor (Bio-Rad, Hemel Hempstead, UK) followed by Superdex 75 gel filtration using a 16 mm ×70 cm column. Two milligrams of *B. g. rhinoceros* venom were mixed with 3 ml of non-reducing rotofor buffer [7M urea, 2M thiourea, 10% (v/v) glycerol and 2.5% (v/v) ampholytes (pI 6–8) in Milli-Q water] and loaded on to the focussing chamber. Isoelectric focussing was performed under cooling setting I (temperature between 4°C and 15°C) with the programmed electric field (150V/2W/20 mA for 15 minutes, 200V/2W/20 mA for 15 minutes, 300V/2W/20 mA for 20 minutes, 350V/2W/20 mA for 20 minutes and 400V/2W/20 mA for 60 minutes). 0.1M orthophosphoric acid and 0.1M sodium hydroxide were used as anode and cathode electrode buffers respectively. Twenty microlitres of separated rotofor fractions were used to analyse the serine protease activity using Arg-AMC and 10 µl were used for SDS-PAGE to analyse the protein separation patterns.

Rotofor fractions with serine protease activity and containing a highly abundant protein with an approximate molecular mass of 36 kDa were pooled and loaded on to a Superdex 75 column with a flow rate of 1ml/min in 0.02M Tris-HCl pH 7.4 and collected as 1 ml fractions. Serine protease activity in 100 µl of selected fractions representing each peak in the chromatogram was measured using Arg-AMC. Each of these fractions (100 µl) were analysed by 10% reducing SDS-PAGE. The purified protein was washed and concentrated by ultrafiltration (30 kDa cut off). Protein concentration was estimated using a Bradford assay [15]. Specific antibodies against the purified protein were raised in rabbit using standard protocols [16].

*Glycosylation detection and deglycosylation*- Twenty micrograms of purified protein were subjected to (i) 10% reducing SDS-PAGE followed by transfer to a PVDF membrane and (ii) glycosylation detection using the ECL glycoprotein detection module according to the manufacturer's protocol. Deglycosylation was achieved by mixing 100 µg of purified protein in 0.02M Tris-HCl pH 7.4 with 5 units of N Glycosidase F and incubating for 10 hours at 37°C.

*Sequencing of purified protein*- Trypsin was used to digest 100 µg of purified protein and the resultant peptides were purified by HPLC and then subjected to Edman degradation. A silver stained reducing SDS-PAGE band of purified serine protease was excised and sequenced by LC-Mass spectrometry (Q-TOF).

*Plasma clotting assay*- The plasma clotting assay was performed as previously described [17]. 100 µg of purified protein and different quantities of thrombin were mixed individually with 500 µl of pre-incubated human citrated plasma at 37°C before adding 200 µl of 0.02M CaCl_2_ to re-calcify the plasma. The mixture was incubated at 37°C in a water bath and the clotting time recorded.

*Fibrinogenolytic assay*- This assay was performed as previously described [18]. 400 µl (5 mg/ml) of plasminogen-free fibrinogen in 0.05M Tris-HCl; 0.1M NaCl, pH 7.8 were mixed with 100 µg of purified protein, and the mixture was incubated at 37°C in a water bath. 25 µl of reaction mix were taken at different time intervals and mixed with an equal volume of Laemmli reducing sample treatment buffer immediately to stop the reaction. Similarly, for the analysis of rhinocerase activity in plasma, 400 µl of human plasma was mixed with 100 µg of rhinocerase and different concentrations of EDTA, and the mixture was incubated at 37°C in a water bath. Fifty microlitres of reaction mix were taken at different time points and mixed with 50 µl of Proteoprep immunoaffinity equilibration buffer and passed through the Proteoprep immunoaffinity albumin and IgG depletion spin columns according to the manufacturer's protocols. The column was washed once with 150 µl of Proteoprep immunoaffinity equilibration buffer and the eluate was mixed with previously collected unbound plasma proteins. Fifty microlitres of the 250 µl of unbound plasma proteins were mixed with an equal volume of Laemmli reducing sample treatment buffer. The bound plasma proteins were eluted with 300 µl of Proteoprep protein extraction reagent type 4 and this was analysed to ensure the absence of any target proteins bound to the column. All samples were analysed by 10% reducing SDS-PAGE.

*Fibrinolytic assay*- Human citrated plasma (500 µl) was mixed with 2 units of thrombin at 37°C in a water bath before adding 100 µl of 0.02M CaCl_2_ and incubated until the formation of a clot. The clot was transferred to another clean test tube and 100 µg of purified protein or buffer were added and incubated at 37°C overnight.

Fibrinolytic activity was also analysed with a fluorescent plasmin substrate [Ala-Leu-Lys-AMC (D-Ala-Leu-Lys-7-amido-4-methylcoumarin) A8171, from Sigma Aldrich, Poole, UK]. Plasmin substrate at various concentrations was incubated with 100 µg of purified protein or venom in 0.1M Tris-HCl pH 7.4 at 37°C. The amount of AMC released was measured at different time points at an excitation wavelength of 366 nm and an emission wavelength of 460 nm. The kinetic parameters were calculated as described for Arg-AMC.

*Kininogenase and plasminogen activator assays*- Kininogenase (kallikrein-like) and plasminogen activator activities were analysed using a fluorescent glandular kallikrein substrate [Ala-Leu-Arg-AMC (D-Ala-Leu-Arg-7-amido-4-methylcoumarin) - V2763] and a fluorescent uPA (urokinase plasminogen activator) substrate [Gly-Gly-Arg-AMC (Z-Gly-Gly-Arg-7-amido-4-methylcoumari hydrochloride) - C9396] respectively (Sigma Aldrich, Poole, UK). Both substrates, at varying concentrations, were incubated with 100 µg of purified protein or venom in 0.1M Tris-HCl pH 7.4 at 37°C separately. The amount of AMC released was measured and the kinetic parameters were calculated as described for Arg-AMC.

*Platelet aggregation assay*- Washed human platelets or platelet rich plasma (PRP) were prepared as described previously by differential centrifugation and rested for 30 minutes at 30°C prior to experimentation [19]. 450 µl of platelets or PRP at a density of 4×10^8^ cells/ml in modified Tyrodes-HEPES buffer (134 mM NaCl, 2.9 mM KCl, 0.34 mM Na_2_HPO_4_, 12 mM NaHCO_3_, 1 mM MgCl_2_, 20 mM HEPES and 5 mM glucose, pH 7.3) were pre-warmed at 37°C for 90 seconds prior to the addition of 100 µg of purified protein or 5 µg/ml concentration of collagen related peptide (CRP) (a collagen receptor glycoprotein VI selective agonist) or 1 U/ml concentration of thrombin in a siliconised cuvette in an optical platelet aggregometer (Chrono-Log Corporation, Havertown, PA, USA) with continuous stirring (1200 g). Platelet aggregation was measured as previously described [20].

*Effect on inhibitors on rhinocerase*- Phenyl methylsulphonyl fluoride (PMSF) at the concentrations of 20, 40, 60, 80 and 100 mM were incubated with 10 U of thrombin or 100 µg of native or deglycosylated rhinocerase at 37°C for 30 minutes. The fluorescent substrate, Arg-AMC was then added to a final concentration of 50 nM and incubated at 37°C. The amount of AMC released was measured as described in the previous section.

## Results

*Serine protease activity of B. g. rhinoceros venom*- To determine the presence of serine proteases in the venom of *B. g. rhinoceros*, Arg-AMC was used due to its specificity for trypsin-like serine proteases. Venom was found to contain serine protease activity similar to trypsin and thrombin (table 1).

**Table 1:** Specificity of rhinocerase towards Arg-AMC.

Activity (Substrate)	Enzyme	*Km* a (µM)	*Vmax* a (s^−1^)	*Kcat* a (s^−1^)	*Kcat/Km* x 10^3a^ (µM^−1^s^−1^)
Serine protease (Arg-AMC)	Trypsin	39.19±1.19	-	114.39±3.6	2919.13±6.7
	Thrombin	60.6±5.92	0.43±0.03	-	-
	Venom	6.66±0.57	0.07±0.003	-	-
	Rhinocerase	80.05±2.02	-	214.52±3.9	2680.15±20.87

a The values are mean ± S.D. (*n* = 3).

100 µg of rhinocerase were incubated with different concentrations of Arg-AMC at 37°C for 7 minutes. The amount of AMC released was measured at an excitation wavelength of 366 nm and an emission wavelength of 460 nm at time intervals up to 7 minutes. 100 µg of *B. g. rhinoceros* venom, 100 µg of trypsin and 10 units of thrombin were also used at the same experimental conditions for comparison. Kinetic parameters were determined from Lineweaver-Burk plots.

**Figure 1:**
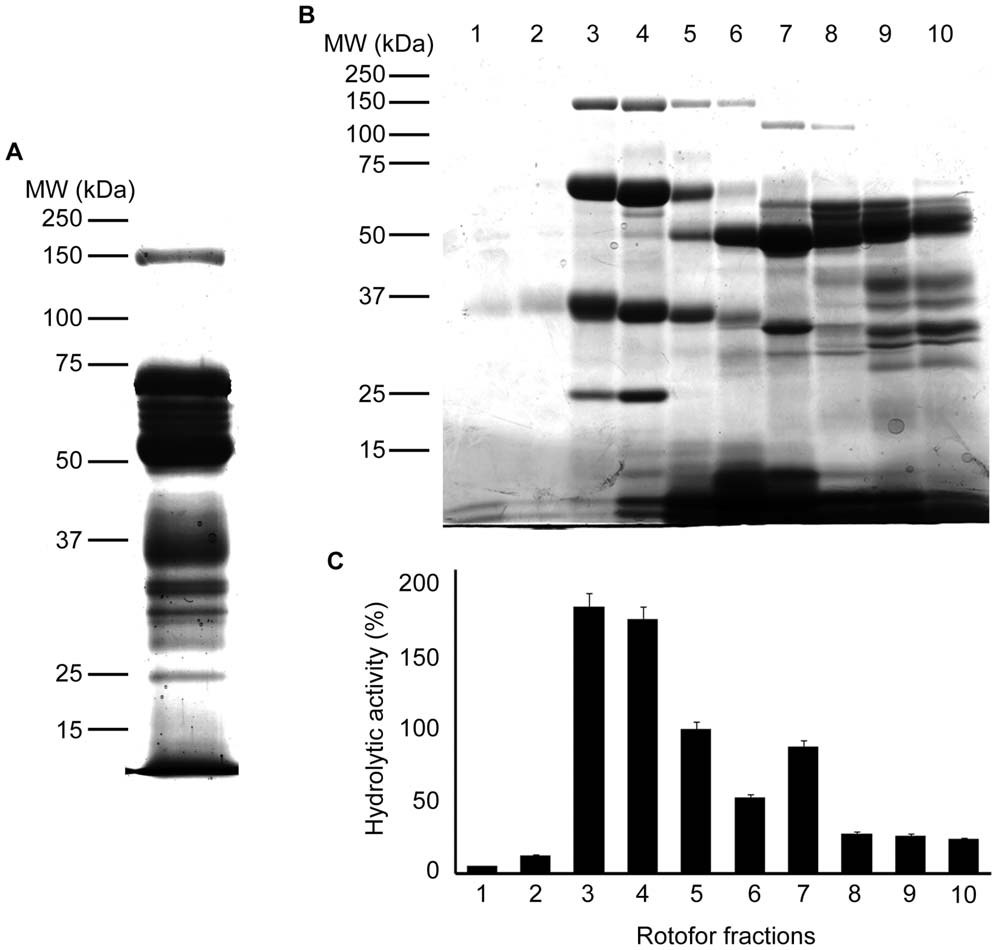
Rotofor based separation of *B. g. rhinoceros* venom proteins. *A.* SDS-PAGE (10%) was run with 50 µg of *B. g. rhinoceros* venom and stained with Coomassie brilliant blue. Several proteins with different molecular weights are present in this venom. *B*. 2 mg of venom were mixed with non-reducing rotofor buffer containing ampholytes with pI 6–8 and separated under non-denaturing conditions. In total 10 fractions (indicated by the numbers at the top of the gel) were collected. 10 µl of each fraction were run in SDS-PAGE (10%) and stained with Coomassie brilliant blue. *C*. 20 µl of each rotofor fraction were used to measure serine protease activity using Arg-AMC fluorescent substrate. The data represents the mean ± S.D. (*n* = 3). The hydrolytic activity measured for fraction 5 was taken as 100%.

*Protein profile of venom*- Fractionation of whole *B. g. rhinoceros* venom using reducing SDS-PAGE yielded 16 visible protein bands of varying staining intensities (figure 1A). The molecular masses of these proteins ranged between 10 kDa and 150 kDa. Eight proteins were visible between 25 kDa and 40 kDa, the molecular weight range where serine proteases have been identified.

**Figure 2:**
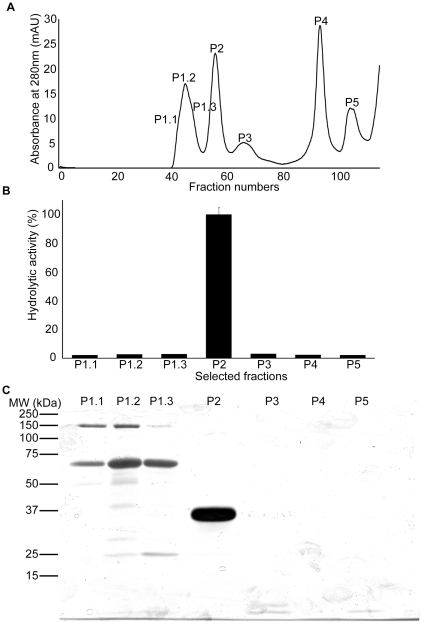
Purification of rhinocerase from rotofor separated venom fractions. *A*. Superdex 75 gel filtration chromatogram obtained during the elution of proteins from rotofor separated venom fractions 3 and 4. The peaks were numbered at the particular fractions which were used for analysis by SDS-PAGE and for analysis of the serine protease activity. *B*. 100 µl of the selected fractions shown in figure *A* were used to measure serine protease activity using Arg-AMC fluorescent substrate. Each bar shows the mean ± S.D. (*n* = 3). The hydrolytic activity measured for P2 was taken as 100%. *C*. 100 µl of the selected fractions indicated in figure *A* were analysed by SDS-PAGE (10%) and silver stained.

*Purification of serine protease* - To purify *B. g. rhinoceros* venom serine proteases size exclusion, ion-exchange and affinity column chromatography were tried either alone or in combination with limited success. We therefore used liquid phase isoelectric focusing which separates proteins based on their isoelectric points. Using an ampholyte range of 6–8 pI (under non-denaturing and non-reducing conditions), venom proteins were well separated (figure 1B) and high serine protease activity observed, which coincided with the presence of two proteins with approximate molecular masses of 36 kDa and 70 kDa in fractions 3 and 4 (figure 1C). These fractions were pooled and loaded onto a Superdex 75 gel filtration column. Five clearly resolved protein peaks (P1, P2, P3, P4 and P5) were identified (figure 2A). Serine protease activity was measured for single fractions with high absorbance in fractions P1.1-P1.3 and P2-P5, and was found to be restricted to peak P2 (figure 2B). Proteins present in these fractions were separated by reducing SDS-PAGE and visualised by silver staining. Peak P2 was found to contain a single protein with an approximate molecular mass of 36 kDa (figure 2C). Peaks P4 and P5 shown on the figure 2B do not show any proteins in the SDS-PAGE (figure 2C) indicating the elution of ampholytes, thiourea and urea present in the rotofor buffer. Consistent with the toxicological literature, we named this new purified venom serine protease ‘rhinocerase’. Based on the Bradford assay, 5 mg of whole *B. g. rhinoceros* venom yielded 1.12 mg of rhinocerase, thus this is a major active component which constitutes approximately 25% of the total protein in this venom.

In order to estimate the serine protease activity of rhinocerase, Arg-AMC was used and the kinetic parameters were calculated for rhinocerase in comparison with *B. g. rhinoceros* venom, trypsin and thrombin (table 1). This analysis showed that the Km value of rhinocerase was higher than that of trypsin and venom which indicates that Arg-AMC is a better substrate for trypsin and venom than rhinocerase. This suggests that the venom might have other serine proteases which cleave Arg-AMC better than rhinocerase. However, Arg-AMC was used throughout the purification process to identify the serine protease activity.

**Figure 3:**
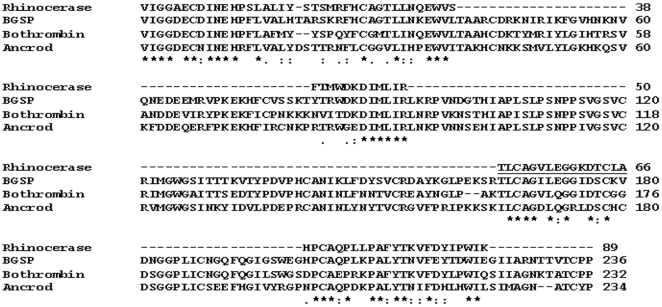
Sequence alignment of rhinocerase with other VVSPs. The rhinocerase sequence obtained by Edman degradation was aligned with BGSP, the only known serine protease sequence from *Bitis gabonica* (NCBI accession number: AAR24534) and two other VVSP sequences; bothrombin (NCBI accession number: P81661) and ancrod (NCBI accession number: AAA49195). The sequence identified by Q-TOF is underlined. ★ indicates conserved residues, : indicates biochemically more related residues and. indicates biochemically less related residues.

*Sequence analysis*- N-terminal sequencing of the undigested protein, together with 4 trypsin digested fragments yielded 89 residues, which comprise approximately 38% of the total sequence based on the mature venom serine protease sequences (230–235 amino acids) reported so far. The resulting sequence was aligned with that of *Bitis gabonica* serine protease 1 (NCBI accession number: AAR24534) (BGSP) [9], the only published serine protease sequence from *B. gabonica* venom and other VVSPs such as bothrombin (NCBI accession number: P81661) and ancrod (NCBI accession number: AAA49195). The sequence alignment (figure 3) shows high similarity between the VVSP sequences, although BGSP and rhinocerase are clearly distinct.

Data obtained by mass spectrometry of rhinocerase were searched using the Mascot database [21] and the closest match was found to be the serine protease Elegaxobin I from *Trimeresurus elegans*. The sequence of a short trypsin-digested peptide was found to be ‘TLCAGVLEGGKD’ and these residues (underlined in figure 3) were also identified using Edman degradation. This suggests that the rhinocerase might be a serine protease, although we have not identified the amino acid present at position 195 (α-chymotrypsinogen numbering), which should correspond to the catalytic serine of serine proteases.

**Figure 4:**
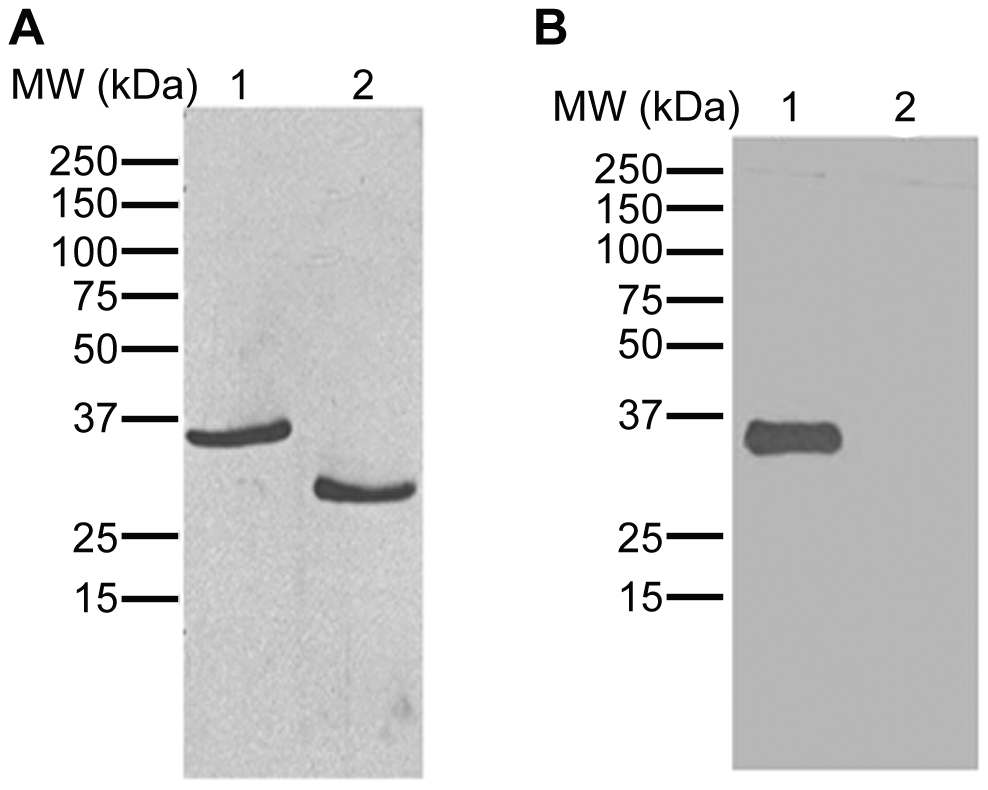
Glycosylation detection on rhinocerase. Deglycosylation was performed by mixing 100 µg of rhinocerase with 5 units of N glycosidase F in 50 µl of 0.02M Tris-HCl pH 7.4. 20 µg of glycosylated (lane1) and deglycosylated (lane2) rhinocerase were run in two separate SDS-PAGE (10%) gels and transferred to two PVDF membranes. *A*. One PVDF membrane was treated with rhinocerase-specific antibody. *B*. The second PVDF membrane was used to detect glycosylation using the ECL glycosylation detection module. Data are representative of three separate experiments.

*Rhinocerase is glycosylated*- Several VVSPs are glycosylated [3], [8], and BGSP, the only serine protease identified from *B. gabonica* venom has three predicted potential N-glycosylation sites. Deglycosylation was performed on rhinocerase using N Glycosidase F and the resulting samples were run in 10% SDS-PAGE along with native rhinocerase. The gel was transferred to a PVDF membrane and subjected to immune blotting with a rhinocerase-specific antibody and glycosylation detection using the glycoprotein detection module. The immuno blot shows that the molecular mass of deglycosylated protein is approximately 31 kDa (figure 4A). Thus, around 5 kDa (14%) of the apparent 36 kDa molecular mass of the rhinocerase was due to glycosylation. Figure 4B confirms that the rhinocerase is glycosylated (lane1) and that all the glycosylation has been removed by the N Glycosidase F (lane2) consistent with complete transition from 36 kDa to 31 kDa as shown in figure 4A. The deglycosylated rhinocerase was tested against Arg-AMC to determine whether it had retained its serine protease activity after deglycosylation, and it was shown to have slightly increased activity compared to the native enzyme.

*Functional characterization*- While some VVSPs have been reported to have more than one function, eg, crotalase has fibrinogenolytic and kininogenolytic functions [22], most of the VVSPs have not been extensively investigated. We therefore sought to characterise rhinocerase for a range of potential functions such as plasma clotting, fibrinogenolytic, fibrinolytic, kininogenolytic, plasminogen activator and platelet aggregating activities.

Plasma was clotted rapidly by all concentrations of thrombin within a few seconds of re-calcification. The venom of *B. g. rhinoceros* made a plasma clot in nine minutes. However, no visible clots were formed with rhinocerase or in the negative control even after incubating overnight. Therefore the weak plasma clotting activity of *B. g. rhinoceros* venom may not be a consequence of rhinocerase activity.

**Figure 5:**
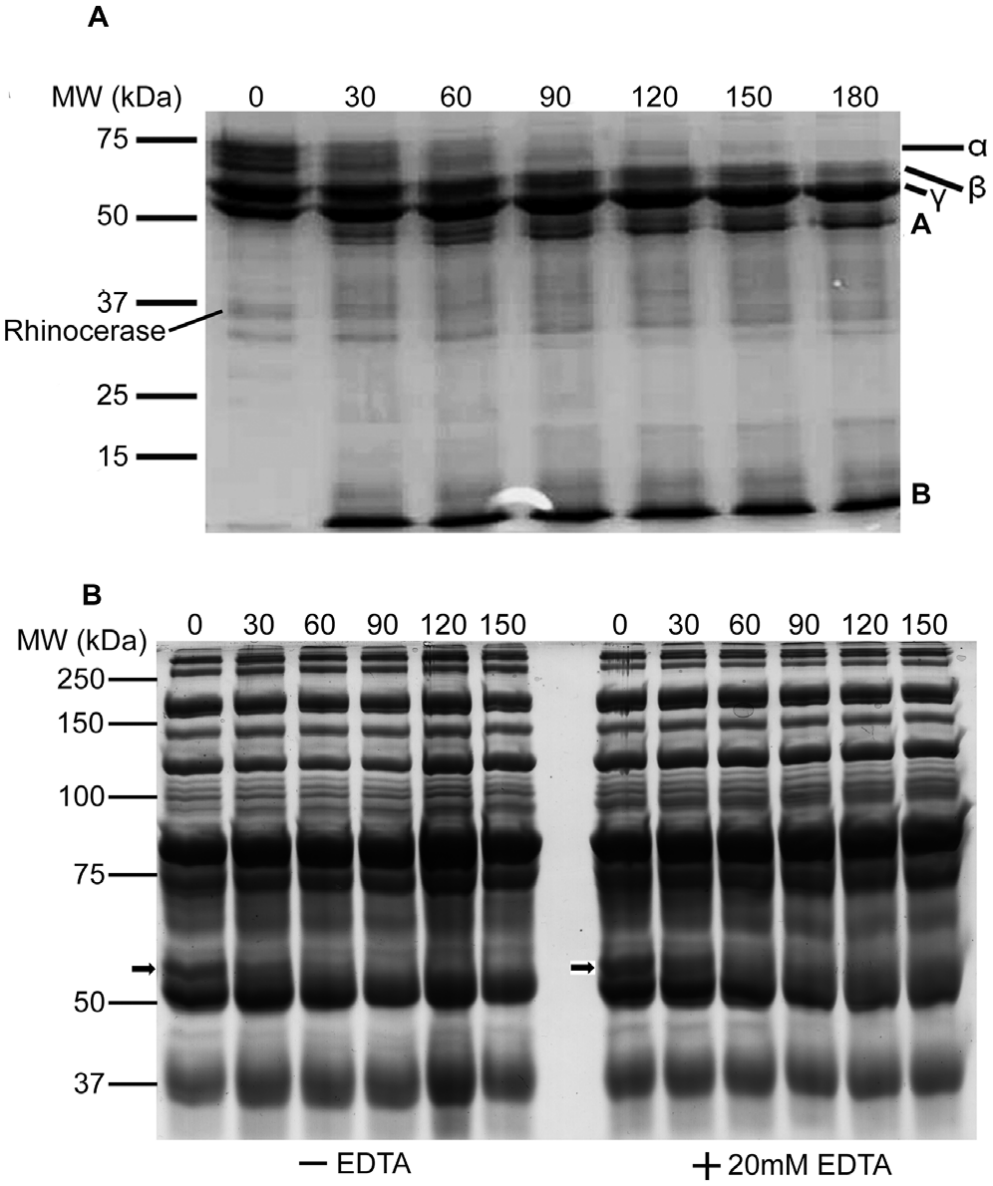
Fibrinogenolytic activity of rhinocerase. Coomassie brilliant blue stained SDS-PAGE gels containing samples taken at different time intervals during the incubation of 100 µg of rhinocerase with 400 µl (5 mg/ml) of plasminogen-free fibrinogen in 0.05M Tris-HCl; 0.1M NaCl, pH 7.8 or 400 µl of human plasma in the presence and absence of 20 mM EDTA. The numbers at the top of each lane represent the time (in minutes) when samples were taken during the digestion. *A*. The degradation profile of plasminogen-free fibrinogen by rhinocerase. α, β and γ represent the three chains of fibrinogen and A and B represent the degradation products. The position of rhinocerase has been labelled on the image. *B*. The degradation profile of fibrinogen present in human plasma after removal of albumin and IgG by Proteoprep immunoaffinity albumin and IgG depletion columns. The position of the α chain of fibrinogen is marked with an arrow. Data are representative of three separate experiments.

Rhinocerase was found to degrade the α and β chains of fibrinogen (figure 5A). The degradation of the α chain was detectable after 30 minutes of incubation with rhinocerase and this chain was degraded completely at 90 minutes. The degradation of the β chain of fibrinogen was detectable after 60 minutes of incubation with rhinocerase and had almost completely degraded at 180 minutes. Protein fragments (labelled A and B) appeared as degradation products. The γ chain of fibrinogen was not degraded by rhinocerase even after 180 minutes. Similarly, rhinocerase was found to degrade the α chain of fibrinogen in human plasma both in the presence and absence of different concentrations (20 mM to 100 mM) of EDTA (figure 5B). The presence of other plasma proteins prevented the degradation of the β chain of fibrinogen from being detected. This clearly suggests that the effect of rhinocerase on fibrinogen is independent of any divalent cations. Interestingly, rhinocerase did not cause the formation of a fibrin clot even after 3 hours. Thus rhinocerase in the venom might be capable of reducing the amount of functional fibrinogen by degrading the α and β chains.

Rhinocerase completely dissolved plasma clots generated by thrombin after incubating overnight. Control clots remained undissolved. This suggests that rhinocerase might have fibrinolytic activity. It was found to be capable of cleaving a plasmin substrate, further supporting the participation of rhinocerase in fibrinolysis (table 2). No activity was seen in control samples.

**Table 2:** Specificity of rhinocerase towards various fluorescent substrates.

Activity (Substrate)	Enzyme	*Km* a (µM)	*Vmax* a(s^−1^)	*Kcat* a (s^−1^)	*Kcat/Km* x 10^3a^ (µM^−1^s^−1^)
Fibrinolytic activity (Ala-Leu-Lys – AMC)	Venom	12.26±0.56	0.32±0.05	-	-
	Rhinocerase	20.29±0.6	-	60.84±0.23	3001.29±99.57
Kininogenolytic activity (Ala-Leu-Arg – AMC)	Venom	14.3±0.33	0.38±0.02	-	-
	Rhinocerase	32.19±0.12	-	104.28±4.77	3239.8±146.28
Plasminogenolytic activity (Gly-Gly-Arg – AMC)	Venom	39.89±1.15	0.37±0.02	-	-
	Rhinocerase	No activity was detected			

a The values are mean ± S.D. (*n* = 3).

100 µg of rhinocerase were incubated with different concentrations of various fluorescent substrates at 37°C for 7 minutes. The amount of AMC released was measured at an excitation wavelength of 366 nm and an emission wavelength of 460 nm at time intervals up to 7 minutes. 100 µg of *B. g. rhinoceros* venom were also used at the same experimental conditions for comparison. Kinetic parameters were determined from Lineweaver-Burk plots.

Rhinocerase hydrolysed the glandular kallikrein substrate, suggesting that it might have a kininogenolytic function (table 2). However, it had no effect on the uPA substrate, though interestingly the *B. g. rhinoceros* venom did hydrolyse this substrate (table 2). This suggests the presence of one or more other serine proteases with plasminogen activator activity in this venom.

**Figure 6:**
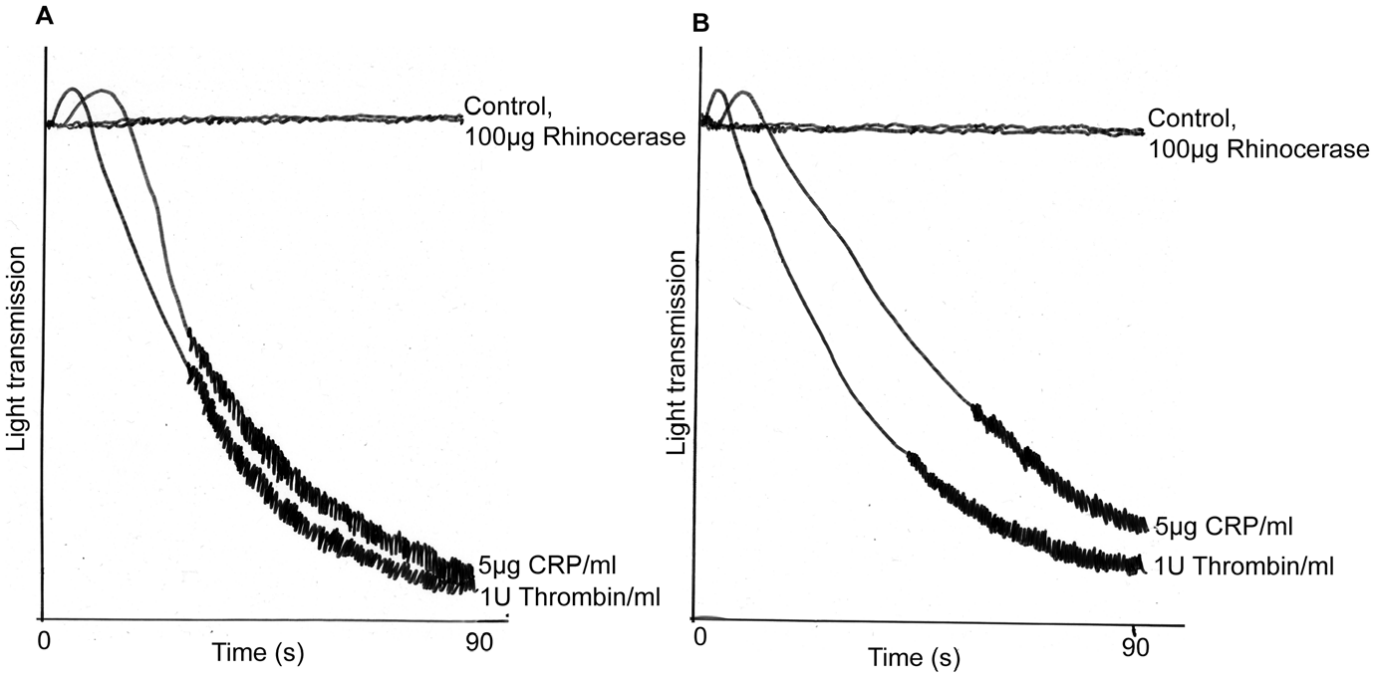
Effect of rhinocerase on platelets. 100 µg of rhinocerase in a 50 µl volume was added to 450 µl of washed human platelets or platelet rich plasma in a siliconised glass cuvette in an optical platelet aggregometer with continuous stirring (1200 g) at 37°C. As positive controls, 5 µg/ml concentration of CRP and 1 U/ml concentration of thrombin were used at same conditions. The traces show nil effect of rhinocerase and aggregation effects of CRP and thrombin on human washed platelets (*A*) and platelet rich plasma (*B*).

Rhinocerase was found to be incapable of stimulating platelet aggregation both in the washed platelets and PRP. This assay was repeated three times using blood platelets or PRP from three different donors but no aggregation was observed, although positive controls (thrombin and collagen related peptide) did aggregate platelets (figure 6).

Together these data indicate that rhinocerase has effects on a variety of substrates and suggests that it could have α and β fibrinogenolytic, fibrinolytic and kininogenolytic activities. Kinetic analysis shows that Ala-Leu-Lys-AMC is a better substrate than Ala-Leu-Arg-AMC as the Km value was lower for the former (table 2). Further to this, the venom of *B. g. rhinoceros* displayed plasma clotting and low levels of plasminogen activator activities. This suggests the presence of additional enzymes, possibly serine proteases with different functions in this venom.

*Effect of inhibitors on rhinocerase activity*- VVSPs have been reported to be sensitive to some but not all serine protease inhibitors. In order to find out the sensitivity of rhinocerase to serine protease inhibitors, we tested the effects of two inhibitors.

**Figure 7:**
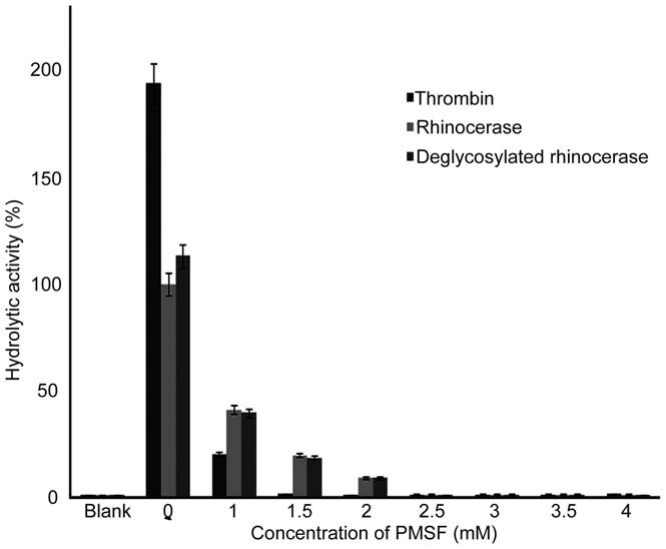
Effect of PMSF on rhinocerase activity. 10 U of thrombin or 100 µg of native and deglycosylated rhinocerase were mixed with different concentrations of PMSF and incubated at 37°C for 30 minutes prior to the addition of Arg-AMC to a final concentration of 50 nM. The amount of Arg-AMC released was measured after 15 minutes of incubation using a spectrofluorometer. Each bar shows the mean ± S.D. (*n* = 3). The hydrolytic activity measured for rhinocerase without any PMSF was taken as 100%.

1.2 mM PMSF is sufficient to inhibit the activity of 10 units of thrombin. 100 µg of rhinocerase activity were inhibited by 2.3 mM PMSF (figure 7). Comparison of thrombin and rhinocerase activity shows that the activity of 100 µg of rhinocerase is equivalent to only 4.05 units of thrombin, and thus we conclude that rhinocerase is less sensitive to PMSF than thrombin. Similarly, the deglycosylated rhinocerase was inhibited by the same concentration of PMSF as native rhinocerase indicating that the glycosylated moieties do not affect the binding of PMSF. A benzamidine affinity column was used to assess the sensitivity of rhinocerase to benzamidine. Rhinocerase was unable to bind the column indicating that benzamidine is not an inhibitor for rhinocerase (data not shown).

## Discussion

A novel and highly abundant serine protease, rhinocerase, has been purified in electrophoretically homogeneous form from the venom of *B. g. rhinoceros*. Rhinocerase is clearly distinct from the serine proteases previously identified in this venom. The apparent molecular mass of rhinocerase was found to be approximately 36 kDa and this did not match with that of any of the other known *B. gabonica* serine proteases. The deglycosylated rhinocerase had a molecular mass of approximately 31 kDa according to its mobility in 10% reducing SDS-PAGE. Three other venom serine protease-containing fractions, all distinct from rhinocerase, have been isolated from *B. gabonica* with approximate molecular masses of 32 kDa based on gel chromatography [10]. Pirkle et al. [12] isolated another serine protease, gabonase, with an estimated molecular mass of 32 kDa based on SDS-PAGE. As these reports did not mention the subspecies of the snake, it is unclear whether they have used *Bitis gabonica gabonica* or *B. g. rhinoceros* as we have used.

The estimated pI of rhinocerase was approximately 6 and again this did not match with the one completely sequenced *B. gabonica* serine protease which has a pI of 8.5 [9]. The N-terminal sequence of rhinocerase was obtained by Edman degradation and this matches with the partial N-terminal sequences of the BG-8 fragment [9] and peptide 21 [11] reported previously. Although not mentioned in their paper, these comparisons suggest that Francischetti et al. [9] also used *B. g. rhinoceros* rather than *B. g. gabonica*.

The molecular masses of venom serine proteases seem to vary mainly as a result of their carbohydrate content, since they all contain a similar number of amino acids i.e. 230-236 [23]. For example, the three serine protease-containing fractions isolated from the venom of *B. gabonica* were reported to have glycosylation corresponding to 20–25% of their native molecular mass [10]. Similarly, a pro-coagulant enzyme from *Agkistrodon rhodostoma* [24] and a kininogenase from *Vipera ammodytes ammodytes* [25] have been reported to have glycosylation corresponding to 18–24% of their native molecular mass. In this study, we have found that rhinocerase has glycosylation corresponding to a slightly lower percentage (14%) of its native molecular mass.

VVSPs possess three main functions that are responsible for their systemic effects in envenomed victims: fibrinogenolysis reduces the functional fibrinogen content; fibrinolysis dissolves the blood clots and kininogenolysis generates kinin and bradykinin which alter blood pressure. The preliminary functional analysis of rhinocerase shows its activity against a variety of substrates and suggests that it might have all three of the above functions. The fibrinogenolytic activity of rhinocerase on human plasma in the presence and absence of a chelating agent suggests that rhinocerase activity is independent of divalent cations present in plasma. The inability of rhinocerase to stimulate platelets indicates that it is unlikely to cleave platelet glycoprotein V or the G protein coupled protease-activated receptors 1 and 4 which are receptors for thrombin on these cells. Other VVSPs with platelet aggregating activities have previously been reported in viper venoms [1], [2]. Detailed functional analysis with biological substrates such as high and low molecular weight kininogen and specific fibrinopeptides would further confirm the functions of rhinocerase. However, to our knowledge, rhinocerase is the first VVSP with the potential to have all three major functions. Clotting activity was previously reported for the purified gabonase in *B. gabonica* venom [12]. Three serine protease containing fractions (E I to E III) isolated from *B. gabonica* venom have showed kinin releasing activity (E I), very low kinin releasing and high fibrinolytic activities (E II) and low clotting activity (E III) [10]. Marsh et al. [26] reported two thrombin-like enzymes from *B. gabonica* venom, one with rapid defibrinating activity and another with a weak defibrinating activity. Some VVSPs from other snakes have been found to have two functions, for example crotalase has both kininogenase and fibrinogenolytic activities [22], and jararacussin [27], habu [28] and kangshuanmeni [29] are examples of serine proteases which have both α and β fibrinogenolytic activities. In common with other VVSPs [2] rhinocerase is less sensitive to PMSF than thrombin and insensitive to benzamidine. The activity of deglycosylated rhinocerase on the synthetic fluorescent substrate and its inhibition by PMSF indicates that glycosylation does not affect the interaction between rhinocerase and small synthetic substrates or inhibitors. The glycosylation of rhinocerase might, however, limit its functions when it is binding to large biological substrates and inhibitors.

The suggested functions of rhinocerase are consistent with the effects of systemic envenoming caused by *B. g. rhinoceros* such as hypotension (e.g. kininogenolytic activity), clotting disorders (e.g. fibrinogenolytic activity) and bleeding disorders (e.g. fibrinolytic activity). It is important to understand that the effects of VVSPs occur in the context of the functional activities of other potent biological venom components including other serine proteases, metalloproteases and phospholipases. Marsh et al. [26] reported that the venom of *B. gabonica* causes rapid defibrination together with widespread haemorrhage, cardiac and pulmonary damage. Rhinocerase does not catalyse plasma clot formation but hydrolyses fibrinogen.

In this study, we have purified rhinocerase and characterised its proteolytic functions. Further functional analysis for factor V, VIII, XIII activator assays, protein C activator assay and capillary permeability assay need to be performed to completely characterise potential minor functions of this enzyme. The complete sequence of this protein also needs to be determined to understand how the multi-functionality relates to the amino acid sequence. Since the specificity and multi-functional nature of rhinocerase can only be understood once its structure is determined, this is a focus of ongoing work. The complete understanding of the sequence, structure and functional relationships of rhinocerase could lead to clinical studies to investigate the potential of this enzyme being used to treat human haemostatic disorders such as heart attack, strokes and hypotension.
